# Inflammatory Pathophysiology as a Contributor to Myeloproliferative Neoplasms

**DOI:** 10.3389/fimmu.2021.683401

**Published:** 2021-06-01

**Authors:** Daniel Arthur Corpuz Fisher, Jared Scott Fowles, Amy Zhou, Stephen Tracy Oh

**Affiliations:** Divisions of Hematology & Oncology, School of Medicine, Washington University in St. Louis, Saint Louis, MO, United States

**Keywords:** myeloproliferative neoplasms, myelofibrosis, cytokines, intracellular signaling, JAK2, NF kappa B (NFκB), tumor necrosis factor (TNF), Ruxolitinib

## Abstract

Myeloid neoplasms, including acute myeloid leukemia (AML), myeloproliferative neoplasms (MPNs), and myelodysplastic syndromes (MDS), feature clonal dominance and remodeling of the bone marrow niche in a manner that promotes malignant over non-malignant hematopoiesis. This take-over of hematopoiesis by the malignant clone is hypothesized to include hyperactivation of inflammatory signaling and overproduction of inflammatory cytokines. In the *Ph*-negative MPNs, inflammatory cytokines are considered to be responsible for a highly deleterious pathophysiologic process: the phenotypic transformation of polycythemia vera (PV) or essential thrombocythemia (ET) to secondary myelofibrosis (MF), and the equivalent emergence of primary myelofibrosis (PMF). Bone marrow fibrosis itself is thought to be mediated heavily by the cytokine TGF-β, and possibly other cytokines produced as a result of hyperactivated JAK2 kinase in the malignant clone. MF also features extramedullary hematopoiesis and progression to bone marrow failure, both of which may be mediated in part by responses to cytokines. In MF, elevated levels of individual cytokines in plasma are adverse prognostic indicators: elevated IL-8/CXCL8, in particular, predicts risk of transformation of MF to secondary AML (sAML). Tumor necrosis factor (TNF, also known as TNFα), may underlie malignant clonal dominance, based on results from mouse models. Human PV and ET, as well as MF, harbor overproduction of multiple cytokines, above what is observed in normal aging, which can lead to cellular signaling abnormalities separate from those directly mediated by hyperactivated JAK2 or MPL kinases. Evidence that NFκB pathway signaling is frequently hyperactivated in a pan-hematopoietic pattern in MPNs, including in cells outside the malignant clone, emphasizes that MPNs are pan-hematopoietic diseases, which remodel the bone marrow milieu to favor persistence of the malignancy. Clinical evidence that JAK2 inhibition by ruxolitinib in MF neither reliably reduces malignant clonal burden nor eliminates cytokine elevations, suggests targeting cytokine mediated signaling as a therapeutic strategy, which is being pursued in new clinical trials. Greater knowledge of inflammatory pathophysiology in MPNs can therefore contribute to the development of more effective therapy.

## Introduction

The *Ph*-negative myeloproliferative neoplasms (MPNs) are chronic myeloid neoplasms, featuring an overproduction of one or more mature non-lymphoid cell lineages. A diversity of clinical presentations may include erythrocytosis, thrombocytosis, and/or myeloproliferation as the primary feature. These can be followed by progression to myelofibrosis as a primary or secondary disease phenotype, cytopenias and bone marrow failure, and/or transformation to secondary acute myeloid leukemia (sAML), for which the prognosis is dismal in the post-MPN setting. Many studies have elucidated that MPNs feature an inflammatory component to their pathophysiology, considered to be secondary to their neoplastic development, based on their relative paucity of somatically acquired driver mutations in inflammatory pathways. Inflammation, particularly in the bone marrow microenvironment, can be a factor in clonal dominance and in the progression of the disease, particularly inasfar as it promotes fibrotic transformation of the bone marrow and suppression of benign hematopoiesis, conferring a competitive advantage to the malignant clone. The inflammatory pathophysiology of MPNs, however, has the potential to be leveraged therapeutically for the development of new treatments and improved future therapy.

## Clinical Aspects of MPNs Suggest Inflammatory as Well as Neoplastic Pathophysiology

Ph-negative MPNs are associated with myeloproliferation, constitutional symptoms, and bone marrow fibrosis, which can also be observed in patients with chronic inflammatory diseases. The activating *JAK2* V617F mutation is the most common driver mutation in MPNs. In polycythemia vera (PV) and essential throbocythemia (ET), the *JAK2* mutation can sustain a condition of chronic inflammation (1–3), explaining the associated constitutional symptoms, thrombosis, and premature atherosclerosis observed in patients with these disorders (4–6). Furthermore, the increases in circulating levels of cytokines, chemokines, and reactive oxygen species (ROS) accumulation in chronic inflammatory states can lead to genetic instability, which may favor the development and progression of neoplasms (7). The current evidence suggests that MPNs are chronic inflammatory conditions in addition to neoplastic disorders, and that both processes contribute to the clinical manifestations and pathogenesis of the disease.

Several studies have suggested an association between autoimmune disorders and hematologic malignancies (8–10). A large population-based retrospective study by Kristinsson et al. (11) of 11,039 MPN patients and 43,550 matched controls found a significantly increased risk of MPN in patients with a prior history of autoimmune disease (11). The study found that individuals with a prior history of any autoimmune disease had a 20% increased risk of developing an MPN. When evaluated by individual autoimmune diseases, the study found a 2 –to 3-fold elevated risk of MPNs among patients with a history of immune thrombocytopenia purpura, Crohn’s disease, polymyalgia rheumatica, giant cell arteritis, aplastic anemia, or Reiter’s syndrome (11). These findings suggest that inflammation could be a predisposing factor for development of MPNs and that the overproduction of inflammatory cytokines associated with autoimmune diseases may play a role in the pathogenesis of MPNs (12).

Inflammation is considered a factor that may promote MPN disease development, progression and/or lead to poorer prognosis overall. The recent findings that clonal hematopoiesis is frequent among adult humans, that *JAK2* V617F is among the most common mutations found in asymptomatic clonal hematopoiesis, and that, impressively, clonal *JAK2* V617F is most frequently acquired in childhood or even in utero, suggest that some biological selective process is necessary to transform asymptomatic *JAK2* V617F mutant clones into overt MPNs (13–17). Chronic inflammation in the bone marrow or in the systemic circulation could contribute to the slow selection for eventually pathogenic mutant clones. Further studies are needed, however, to elucidate the specific relationships between inflammatory disorders and MPNs.

A notable feature of MPNs is their diversity of disease phenotypes. MPNs may present as ET, PV, or PMF, often following years to decades of asymptomatic clonal hematopoiesis (13, 14, 16, 17). The malignant clones in the vast majority of MPN patients harbor mutations in *JAK2*, calreticulin (*CALR*), or *MPL* (18). Nearly all PV clones are *JAK2* mutant, however, while ET and PMF clones may harbor mutations in any one of *JAK2, CALR*, or *MPL*. *JAK2* mutant clones can give rise to any of the three disease phenotypes. MPN clones can differ in their propensity to induce inflammatory pathophysiology, which can, in turn, affect their disease phenotype. It has been observed that ex vivo erythroid cell colonies derived from patients with either ET or PV differed in their propensity to harbor elevated interferon γ and STAT1 directed gene expression, which was more prevalent in ET versus PV derived colonies (19). This indicated that inflammatory signaling might alter disease pathophysiology even in the context of a common driver mutation.

*JAK2* mutant homozygosity is substantially more common in PV and MF than in ET (20, 21). It has also been associated with more severe symptoms and increased risk of cardiovascular events in PV (22). In ET and PV, acute phase inflammatory proteins such as high sensitivity (hs)-CRP and pentraxin 3 (PTX-3) were found to significantly correlate with *JAK2* V617F allele burdens of greater than 50% (23–25). Hs-CRP levels were shown to be increased in MPN patients compared to normal controls, and independently associated with shortened leukemia free survival in myelofibrosis (MF) patients (26). Increased levels of hs-CRP were associated with an increased risk of thrombosis, although conversely, high PTX-3 levels were associated with a lower rate of thrombosis (23). Importantly, however, *JAK2* V617F allele burdens of greater than 50% in MF patients have also been associated with favorable responses to ruxolitinib (27), suggesting that MPN patients with elevated hs-CRP or PTX-3 may benefit from aggressive JAK inhibitor therapy.

Consistent with evidence of elevated inflammation, *JAK2* mutant homozygosity in PV or ET increases risk of transformation to MF (28). In contrast to mutant *JAK2*, mutant *CALR* and *MPL* alleles almost never develop homozygosity (29, 30). The contribution of *JAK2* mutant homozygosity to the inflammatory pathophysiology of MPNs remains obscure, but might contribute to differences between outcomes of *JAK2* mutant ET or MF patients versus others. In PMF, survival (either overall or leukemia free) is inferior in the *JAK2* mutant patient population (as compared to *MPL* or *CALR* mutant patients; although triple negative, or 3N, PMF shows even worse survival) (31, 32). The observation of typically greater NFκB activation in hematopoietic stem and progenitor cells (HSPCs) from *JAK2* mutant MF patients (33) is suggestive of more severe inflammation, which may in turn contribute to poor outcomes. More widespread expression of mutant *JAK2* versus *MPL* or *CALR* (34) among hematopoietic cells may contribute to greater inflammation in *JAK2* mutant patients as well.

Despite evidence of inflammatory pathophysiology contributing to poor outcomes in MPNs, specifically to the development of secondary MF and thromboses (3, 5, 23, 25, 26, 28, 35, 36), other factors are known to influence outcomes in MPNs, some of which may have divergent effects from those caused by inflammation. Epigenetic and RNA splicing related mutations are well recognized as predictive of adverse outcomes in MPNs (18, 31). It is notable that low, rather than high, *JAK2* V617F allele burdens at diagnosis have been correlated with shortened leukemia-free survival in PMF (37, 38). Low *JAK2* V617F allele burdens at diagnosis may be associated with anemia and cytopenias in MF (38), and possibly with epigenetic mutations producing prognoses more similar to those of triple negative, or 3N, PMF (32, 39). In PV, low *JAK2* V617F allele burdens at diagnosis are also common in younger patients who frequently present with thrombotic events (40, 41).

## Cytokines Are Elevated in All Chronic Phase MPNs in Comparison With Healthy Aged Individuals

Multiple studies that investigated cytokine levels in MPNs have now produced data showing widespread cytokine elevations in ET, PV, and PMF, and correlations with disease features and outcomes (**Tables 1** and **2**). These studies have identified not only the cytokine elevations most associated with particular disease phenotypes, but also with blast transformation of a chronic phase MPN, or with prognosis. The wide variation in the studies in terms of technology, disease subtype, and specific cytokines measured, poses a challenge for hypothesis generation. Regardless, underlying concepts are coming into focus, namely that: 1) elevated cytokines are observed in MPN patients of all subtypes compared to healthy individuals; 2) the elevated cytokine profiles between subtypes appear distinct in composition or magnitude albeit overlapping among different MPN diagnoses on the level of individual cytokines. Evidence of elevated cytokine levels in MPN patients began to emerge over 30 years ago, with studies relying predominantly on ELISA and/or semiquantitative real time PCR of a small collection of targets. The development of multiplex array-based and single cell technologies in recent years has allowed researchers to interrogate large panels of cytokines to further illuminate the connections between inflammatory cytokines and other pathophysiologic features of myeloid malignancies.

**Table 1:** Selection of inflammatory cytokines elevated in plasmas of MPN patients of all disease stages.

**1^st^ Author**	Pardanani (42)	Tefferi (43)	Vaidya (44)	Kalota (45)	Pourcelot (46)	Cacemiro (47)	Fisher (48)	Fowles (41)
**journal**	Am J Hematol	J Clin Oncol	Am J Hematol	Clin Cancer Res	Exp Hematol	Hematol Transfus Cell Ther	Leukemia	Leukemia
**year**	2011	2011	2012	2013	2014	2019	2019	2019
**EGF**	ns	ns	HD > PV; PMF > PV	*-*	*-*	*-*	*-*	*-*
**b-FGF**	ns	ns	PMF > PV	*-*	*-*	*-*	*-*	*-*
**G-CSF**	ns	PMF > HD	ns	MF > HD; PV > HD	*-*	*-*	*-*	ns
**GM-CSF**	MF > HD	ns	PV > HD; PV > PMF	MF > HD; PV > HD	PV > HD; ET > HD	MPN > HD; PV > ET	ns	*-*
**HGF**	MF > HD	PMF > HD	PV > HD	*-*	PV > HD; ET > HD	*-*	*-*	*-*
**IFN-α**	ns	PMF > HD	PMF > PV	MF > HD; PV > HD	*-*	MPN > HD	*-*	*-*
**IFN-γ**	MF > HD	HD > PMF	PV > PMF	MF > HD; PV > HD	PV > HD; ET > HD	PMF > ET	ns	PV > HD
**IL-4**	MF > HD	ns	ns	*-*	PV > HD; ET > HD	MPN > HD; PV > ET	ns	*-*
**IL-5**	ns	ns	PV > HD	*-*	*-*	MPN > HD	ns	*-*
**IL-6**	MF > HD	PMF > HD	PV > HD	MF > HD; PV > HD	PV > HD; ET > HD	MPN > HD	MF > HD	PV > HD
**IL-8**	MF > HD	PMF > HD	PV > HD	*-*	PV > HD; ET > HD	*-*	*-*	ns
**IL-10**	MF > HD	PMF > HD	PMF > PV	*-*	PV > HD; ET > HD	MPN > HD	MF > HD	*-*
**IL-12**	MF > HD	PMF > HD	PV > HD; PMF > PV	*-*	PV > HD; ET > HD	MPN > HD; PMF > ET; PV > ET	ns	*-*
**IL-13**	MF > HD	PMF > HD	PV > HD	*-*	*-*	*-*	ns	*-*
**IL-15**	MF > HD	PMF > HD	PV > HD	*-*	*-*	*-*	ns	*-*
**IL-16**	ns	*-*	*-*	*-*	*-*	*-*	MF > HD	*-*
**IL-17**	MF > HD	ns	ns	*-*	*-*	PMF > HD; PMF > ET	ns	*-*
**IL-1RA**	MF > HD	PMF > HD	PV > HD; PMF > PV	*-*	*-*	*-*	*-*	*-*
**IL-1β**	MF > HD	PMF > HD	PMF > PV	*-*	ns	MPN > HD	ns	*-*
**IP-10**	MF > HD	PMF > HD	PV > HD; PV > PMF	*-*	*-*	PMF > ET	ns	PV > HD
**MCP-1**	MF > HD	PMF > HD	PV > HD	*-*	PV > HD; ET > HD	ns	ns	*-*
**MIG**	MF > HD	PMF > HD	PV > HD; PV > PMF	*-*	*-*	*-*	*-*	*-*
**MIP-1α**	MF > HD	PMF > HD	PV > HD; PV > PMF	*-*	*-*	MPN > HD	ns	PV > HD
**MIP-1β**	MF > HD	PMF > HD	PV > HD; PV > PMF	MF > HD; PV > HD	*-*	PV > HD; ET > HD	ns	*-*
**RANTES**	MF > HD	ns	HD > PV; PMF > PV	MF > HD; PV > HD	*-*	ET > HD; PV > ET	*-*	*-*
**sIL-2R**	MF > HD	PMF > HD	PMF > PV	*-*	*-*	*-*	*-*	PV > HD
**TNFRII**	*-*	*-*	*-*	*-*	*-*	*-*	*-*	*-*
**TNF-α**	MF > HD	PMF > HD	ns	MF > HD; PV > HD	PV > HD; ET > HD	MPN > HD	ns	PV > HD
**VEGF**	MF > HD	PMF > HD	PV > HD; PV > PMF	*-*	PV > HD; ET > HD	*-*	MF > HD	ns

MF, myelofibrosis; PMF, primary myelofibrosis; HD, healthy donor; PV, polycythemia vera; ET, essential thrombocythemia; MPN, myeloproliferative neoplasms; ns, no significant differences between sample types; “>“, first sample type is significantly elevated compared to second sample type; “-”, not in assay.

**Table 2:** Cytokine correlations with disease features and outcomes in MPNs.

	reduced survival	MF trans-formation	AML trans-formation	leukocytosis	HCT/Hgb	transfusion dependence	thrombo-cytosis	thrombosis	splenomegaly	vascular complications	*JAK2* burden	Age	sex (M)
**GM-CSF**	PV (44)						↓PV (44)			↓PV, ↓ET (46)			
**HGF**				PMF (43); PV (44); PV (49)	PV (49)				PMF (43)	↓PV (44)	PMF (43)		
**hs-CRP**	MF (23, 25)		MF (23)					PV& ET (23, 24)			PV& ET (23, 24), PMF (25)	PMF (25)	
**IFN-α**	PV (44)						PV (44); PMF (47)						
**IL-1β**	PV (44)	PV (44)		PV (44)									
**IL-2**		PV, ET (50)	MF (51); PMF (50)	PV (44)									
**sIL-2R**	PMF* (43); MF* (52)	PV, ET (50)	MF (51); PMF (50)	PMF* (43); PMF (35)	↓JAK2 mut PMF (35)	PMF* (43); MF (52)	↓JAK2mut PMF (35)		MF (52); JAK2 mut PMF (35)		PMF* (43)	PV (41)	PMF (43)
**IL-4**	PV (44)				PV (46); ↓PV (47)					↓PV (44)			
**IL-5**	PV (44)	PV (44)		↓PV (47)									
**IL-6**		PV, ET (50); PV (44)	PMF (50)	PV (46)	↓PV (44)						PMF (43)	PV (41)	
**IL-8**	PMF* (43); MF* (52)		PMF (43)	PMF* (43)									PMF (43)
**IL-10**	PV (44)	PV (44)				PMF (43)							
**IL-12**		PV (44)			PV (44)	PMF* (43)				↓PV, ↓ET (46)	PMF (43)		
**IL-15**	PMF* (43)	PV (44)											PMF* (43)
**IP-10**	PMF (43)	PV (44)		PMF (43)			↓PMF (47)				PMF (43); PMF (47)	PMF (43); PV (41)	
**MCP-1**	PV (44)				PV (46)	PMF (43)			PV (44)				
**MIG**									PMF (43)		PMF (43)		PMF (43)
**MIP-1α**	PV (44)				↓PMF (47)	PMF (43)							PMF* (43)
**MIP-1β**	PV* (44)												
**TNF-α**	PV (44)			PV (46)	PV (46)		ET (47)				MPN (53)	PV (41)	

MF, myelofibrosis; AML, acute myeloid leukemia; HCT, hematocrit; Hgb, hemoglobin; PV, polycythemia vera; ET, essential thrombocythemia; PMF, primary myelofibrosis; MPN, myeloproliferative neoplasm; * significant in multivariate analysis; ↓, lower cytokine levels is associated with disease feature; superscript numbers refer to reference number in bibliography.

In studies that correlated cytokine levels from PV and ET samples with disease features, IL-2, s-IL-2R, and IL-6 correlated with MF transformation from both PV and ET (50). Additionally, CRP correlated with thrombosis and JAK2 V617F burden in a combined PV/ET cohort (23). In ET where overproduction of platelets is a main feature, a study observed a correlation with thrombocytosis and TNF levels (47).

Patients with primary myelofibrosis (PMF) have been shown to share both clinical symptoms and laboratory abnormalities with patients with systemic inflammatory response syndrome, such as elevation in the erythrocyte sedimentation rate, C-reactive protein (CRP), IL-1β, IL-6, IL-8, and TNF (54). High plasma levels of IL-6 and IL-8 were found to be significantly associated with severity of constitutional symptoms in PMF patients by Tefferi et al. (43) Elevations of IL-2, and sIL-2R, Il-6, IL-8, and β2-microglobulin, have been associated with blast transformation of CML or *Ph-* MPNs, based on several studies (43, 50, 51, 55). IL-8, also known as CXCL8, is a CXC family cytokine found to be expressed by HSPC in MF (48) and *de novo* AML (56) patients. High levels of IL-8 were associated with both poor overall and leukemia-free survival, and transformation to sAML, when assayed in blood plasma from MF patients (43). IL-8 has thereby been hypothesized to be a potential surrogate for CD34+ cell burden in MF, and hence a possible risk marker for incipient transformation to sAML (48). Tefferi et al. also found plasma IL-12, IL-15, IP-10, and circulating IL-2R, to be independent markers of poor survival, as well as IL-8 (43).

The prognostic potential of cytokines in PV was addressed in 2012 by Vaidya et al., revealing a different profile from what was observed for MF (44). Thirty cytokines were measured from plasma samples from 65 patients using the same Luminex technology as in the preceding study by Tefferi et al. for MF (43). A univariate analysis showed association of multiple cytokines with inferior overall survival in PV, but CCL4/MIP-1β alone remained associated in a multivariable analysis. Fibrotic transformation was associated in a univariate analysis with elevations in IL-1β, IL-5, IL-6, IL-10, IL-12, IL-15, IL-17, and IP-10 (44).

Verstovsek et al. showed that levels of several cytokines from 25 patients enrolled in the Phase 1-2 clinical trial of ruxolitinib for MF, before and 28 days-post treatment decreased significantly (57). Reductions of CRP, IL-1RA, CCL4/MIP-1β, TNF, and IL-6 were associated with decrease in the composite symptom score of patients (57). Pardanani et al. identified in MF patients from two different clinical trials of pomalidomide for MF-associated anemia, that patients with high levels of sIL-2R, IL-8, IL-15, MCP-1, and VEGF at baseline had significantly lower rates of anemia response to treatment (42).

Inflammation has been shown to increase with age, consistent with changes in hematopoiesis observed in healthy aging. Although much less frequently, MPN can occur at younger ages. In 2019, a study by the authors examined the relationship of age in PV patients with both inflammation and genomic mutation profile (41). Comparing plasma from 16 young PV patients (age ≤ 45 years) with 12 old PV patients (age ≥ 65 years) that were all within 1.5 years of diagnosis, the same cytokines were significantly elevated compared to age-matched healthy donors (10 young and 7 old). When comparing the fold change based on their respective age-matched donors, old PV patients exhibited an exacerbated elevation of cytokines compared to young PV patients. Overall mutational burden increased with age as expected, and secondary non-*JAK2* driver mutations were found in 9/10 old PV patients, in contrast with young PV patients, where *JAK2* V617F appeared to be the only disease-related mutation present (41). Together, this data suggests two important concepts for MPN pathophysiology: First, that PV patients exhibit elevated inflammatory cytokine production regardless of age. Second, that the more pronounced elevation of cytokines observed in old PV patients could be attributable to age and/or the presence of secondary mutations. It is important to note that in this study no predominance of non-JAK2 mutations preceding *JAK2* V617F was observed. This suggests that the increase in MPN-related mutational burden might not be a mere function of age, but also a result of the *JAK2* mutation itself, as suggested by findings that *JAK2* V617F may indirectly promote genomic instability (58).

Taken together, these studies provide evidence that inflammatory cytokine levels are elevated in MPNs compared to healthy counterparts, that this elevation can be seen even among relatively young MPN patients, and that other signaling pathways or factors may be involved besides JAK-STAT signaling. Furthermore, plasma cytokines levels in MF and sAML patient samples, assayed by V-PLEX human cytokine 30-plex assay, demonstrated that sAML cytokine levels were similar to MF levels (48). Therefore, the pattern of elevation of plasma cytokines observed in chronic MPNs persists in sAML, despite the extremely altered cellular composition of AML versus chronic MPNs.

## Hyperactivation of JAK2 or MPL Causes Myeloproliferation, Cytokine Production, and Myelofibrosis

**Figure 1:**
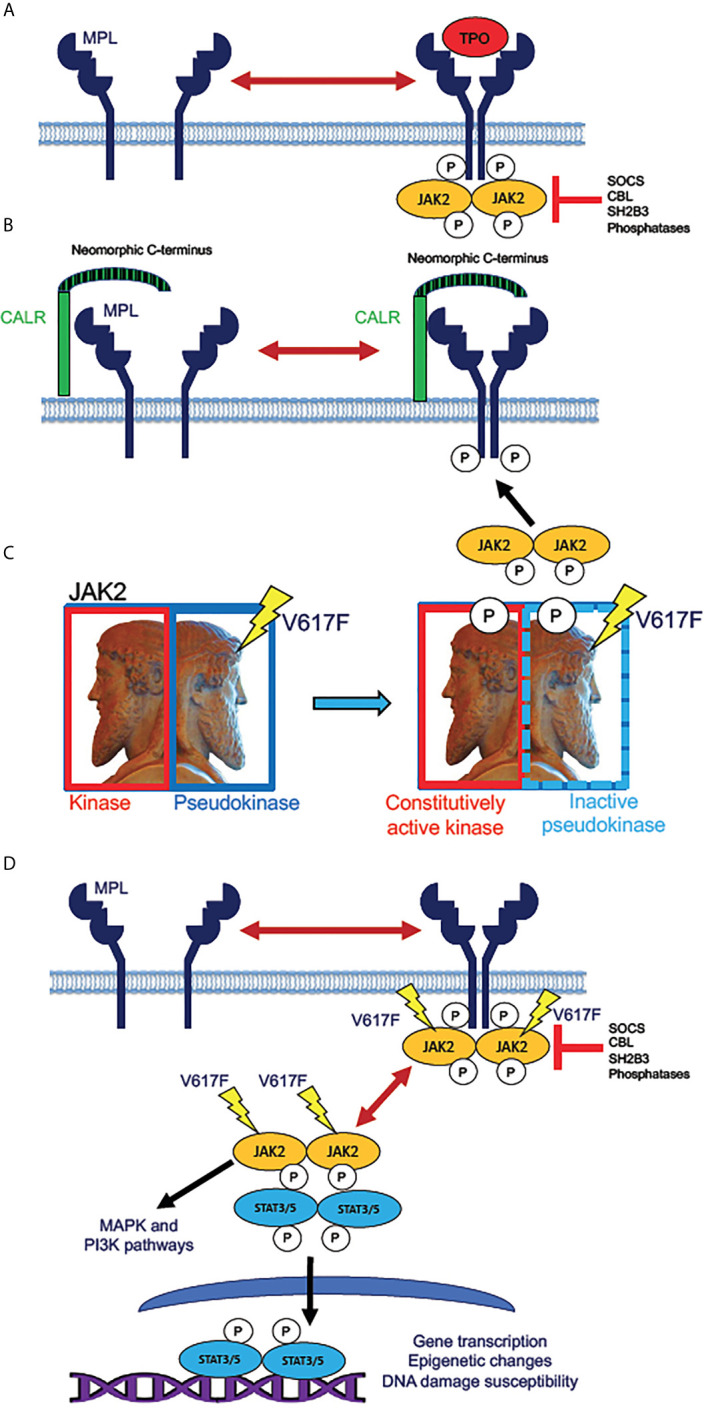
Mechanisms of JAK2 activation by MPN driver mutations. **(A)** Normal mechanism for receptor activation of the human thrombopoietin (TPO) receptor MPL (myeloproliferative leukemia proto-oncogene). MPL, expressed in HSPC and megakaryocytic lineage cells, exists in an equilibrium between inactive monomers and active homodimers. Binding of the monomeric ligand TPO stabilizes dimer formation, allowing phosphorylation of the receptor by dimeric JAK2 tyrosine kinase. This step initiates intracellular signaling downstream of active JAK2. The active receptor-kinase holocomplex is subject to inhibition by several inhibitor molecules, notably SOCS (suppressor of cytokine signaling) family proteins, tyrosine phosphatases, and the adaptor protein SH2B3 (also known as LNK, lymphocyte linker protein). Mutations in genes encoding these various inhibitory molecules are rare driver mutations in MPNs, which lead to JAK2 hyperactivation by removal of physiologic inhibition. **(B)** Activation of JAK2 signaling by mutant CALR. The single-pass transmembrane protein CALR (calreticulin) is a calcium-binding chaperone protein that normally recycles between plasma and intracellular membranes in the secretory pathway. Mutated CALR acquires a neomorphic C-terminus (depicted as striped), which is capable of binding to and dimerizing MPL, consequently producing signaling-active MPL homodimers, which recruit and activate JAK2, in the absence of TPO binding. *CALR* mutations in MPN are therefore hypothesized to have similar effects to activating *MPL* W515L/K mutations, which are present in roughly 10% of ET and MF patients. **(C)** Effect of the *JAK2* V617F mutation. JAK2 (Janus kinase), depicted as the ancient Roman god Janus (sculpture in the Vatican Museum, Rome), contains homologous kinase and pseudokinase domains, the pseudokinase being inhibitory to the kinase. V617F mutation in the pseudokinase domain inactivates the inhibition, producing a constitutively active (autophosphorylated) kinase. **(D)** Direct activation of JAK/STAT signaling by active JAK2. Mutant JAK2 (typically V617F) dimerizes MPL and other cytokine receptors, rendering the receptor active even in absence of a bound ligand. The active receptor-bound JAK2 phosphorylates STAT3 and STAT5 homodimers, which then translocate to the nucleus to activate transcription. Unlike *MPL* and *CALR* mutations, *JAK2* mutations enable constitutive JAK2 activity even in cells that do not express *MPL*. Mutant JAK2 has been shown to promote epigenetic changes and increase the potential for unrepaired DNA damage. Active JAK2 collaterally activates MAP kinase (MAPK) and PI3 kinase (PI3K) signaling pathways independently of STAT3 and STAT5.

The *Ph*-negative myeloproliferative neoplasms (MPNs), polycythemia vera (PV), essential thrombocythemia (ET), primary or idiopathic myelofibrosis (PMF), and secondary myelofibrosis (MF, secondary to PV or ET), share a common etiology in hyperactivation of the kinase JAK2 in the hematopoietic stem and progenitor cell (HSPC) compartment of a malignant clone. Genomic studies of MPNs have revealed that nearly all cases of PV harbor mutations in the gene encoding JAK2 itself, with the specific hyperactivating *JAK2* V617F mutation being present in over 90% of cases of PV, and slightly over half of all studied cases of ET and PMF (31, 40, 59, 60). Roughly 5-10% of ET and MF patients harbor mutations in the *MPL* gene, which encodes the cell surface receptor for thrombopoietin (TPO), which signals intracellularly by binding to and activating JAK2 kinase. A greater portion, 30-35% of ET and MF patients, harbor mutations in calreticulin (*CALR*), a multifunctional protein typically resident in the endoplasmic reticulum, but occasionally exposed at the cell surface (29, 30, 34). Mutant versions of CALR protein contain a neomorphic C-terminal domain (resulting from a frameshift in the genomic DNA sequence) that complexes MPL molecules at the cell surface and predisposes them to constitutive signaling activity (61). Therefore, in almost all cases of MPNs, the malignant clone harbors a mutation conferring a constitutive activation of JAK2 activity in the HSPC population, and throughout derivative malignant hematopoiesis (**Figure 1**). It is not clear if this is true in the case of every MPN patient, as there exist rare triple negative (3N) cases lacking mutations in any of the *JAK2*, *MPL*, or *CALR* genes. Some of these 3N cases, however, have been found to harbor mutations in genes such as *CBL* and *SH2B3/LNK* (62, 63), which confer enhanced JAK2 activity by removal of inhibition; hence it is likely that all *Ph*-negative MPN cases include constitutive JAK2 activity in the malignant clone (60). JAK2 inhibition, most frequently with ruxolitinib, the first JAK inhibitor approved for treatment of MF, remains the best available therapy and standard of care for many MF patients today, and provides clinical benefits to selected PV (and some ET) patients as well (64–70).

The identification of disease-driver mutations in *MPL* and *CALR*, in addition to those in *JAK2*, confers clues to the molecular pathophysiology underlying the diversity of MPN phenotypes. *MPL* mutations in MPNs are activating mutations that facilitate activation of JAK2 kinase by MPL receptor. The *MPL* gene is expressed in HSPCs, including stem cells and early myeloid progenitors, in all stages of the megakaryocytic lineage, and in a subset of monocytic lineage cells, some of which may be fibrogenic (71). These cells respond to TPO by activating JAK2, which phosphorylates the transcription factors STAT3 and STAT5, to transcriptionally active forms capable of mediating cell-type-specific transcriptomic profiles (72, 73). *MPL* and *CALR* mutations are notably not observed in PV, presumably because the *MPL* gene is not expressed in erythroid progenitors, the proliferation of which is normally driven by erythropoietin rather than TPO. A mouse model of Mpl hyperactivity *in vivo*, however, is sufficient to produce bone marrow fibrosis that resembles that observed in MF: Tpo treatment of mice induces bone marrow fibrosis, and this has been used as a model to study this pathophysiologic process, albeit in the absence of a malignant clone (74). This stands in contrast to mouse models expressing MPN-derived mutations in *JAK2* and *CALR* homologs at physiologic levels, which exhibit phenotypes resembling PV or ET, with little if any bone marrow fibrosis (75). In humans, progression of PV or ET to secondary MF often occurs over a number of years greater than the lifespan of a mouse (28).

The specific pathophysiology of myelofibrosis has long been hypothesized to be reactive, since the bone marrow stromal cells are non-malignant, with an important role played by cytokines secreted from malignant cells (54, 76). In the Tpo induced model of bone marrow fibrosis, the cytokine transforming growth factor beta (Tgf-β) was found to be essential to the development of the fibrotic phenotype (74). The essential lesson from this study was that Tpo-responsive cells (hence, Mpl expressing) directed bone marrow fibrosis non-cell-autonomously *via* the production of another cytokine. This illustrates the inflammatory hypothesis of MPN pathophysiology, a hypothesis that has been applied to other cancers as well: namely, that inflammation, particularly *via* inflammatory cytokines, is a major driver of disease phenotype.

Furthermore, the hyperabundance of inflammatory mediators that is present in myeloid neoplasms is not confined to circulating cytokines. Cell-contact-mediated inflammatory activation is certainly also a feature of these diseases. In the case of MF, cell-contact-mediated inflammatory activating ligands, such as FAS and the endogenous toll-like-receptor ligands S100A8 and A100A9, have been found to be upregulated at the gene expression level in the malignant CD34-expressing HSPC population (48, 77). This is important because, in MPNs, the HSPC population is not only the disease-propagating population from which the malignant clone initially arises, but also because it is a malignant population that remains present over the course of years in the chronic (ET or PV) phase of the MPN, and hence must be at least partly responsible for effecting phenotypic transformation, such as from ET or PV to MF (78). In MPNs, the entire compendium of malignant pathophysiology must ultimately originate from the stem cell population.

In contrast to the dependence of MPN pathophysiology on the actions of malignant HSPCs, MPNs are also pan-hematopoietic diseases, involving the entire hematopoietic system of the patient, as well as the niche environments supporting hematopoiesis, such as the bone marrow and splenic stroma. Prominent features of MF pathophysiology include HSPC mobilization from the bone marrow, and consequent extramedullary hematopoiesis in the spleen (and occasionally in the liver), producing marked splenomegaly. Therefore, the roles of multiple hematopoietic cell niches need to be considered in the total extent of pathophysiology of MPNs, as components of the affected hematopoietic system.

## MF Cytokine Overproduction Depends on Signaling Abnormalities Beyond JAK-STAT

MPN pathophysiology is predominantly dependent on the common feature of hyperactivation of JAK2 kinase. Therefore, it is of interest to determine to what extent the inflammatory features of MPNs are directly, or indirectly, dependent on JAK2 activity. This is also important because JAK2 inhibition, as a therapeutic modality for MPNs, does not greatly or reliably reduce the malignant clonal burden or extent of bone marrow fibrosis (68, 79, 80). Therapeutic JAK2 inhibition with ruxolitinib was observed to result in reductions of multiple plasma cytokines in MF patients within the first month of treatment (57). Ruxolitinib treatment of MF patients for periods from one month to over a year, however, was observed not to revert plasma cytokines to the low levels seen in healthy control plasmas (48). Therefore, ruxolitinib can be said to provide a partial, but incomplete, reduction of inflammatory pathophysiology in MF. This conclusion raises the important question of whether longstanding MPN pathophysiology has activated signaling pathways that cannot be restored to their normal state by ameliorating the primary signaling defect of JAK2 hyperactivation. Multiple cytokines significantly elevated in MF plasmas activate downstream signaling other than *via* JAK-STAT. These included TNF, which is known to activate multiple signaling pathways, including pro-apoptotic signaling and the canonical NFκB pathway (81). Other studies identified pathologic production of TNF in ET, PV, and sAML, as well as in *de novo* AML, implying non-JAK/STAT signaling hyperactivations almost certainly occur across the spectrum of MPNs (33, 41, 53, 82).

Our group has utilized mass cytometry (CyTOF) to survey both intracellular signaling in MF and sAML (33) and cytokine production in human MF patient blood cells *ex vivo* (48). A survey of intracellular signaling identified frequent elevations of MAP kinase, PI3 kinase, and NFκB pathway signaling markers in MF and sAML patients HSPCs, and in some other myeloid populations such as monocytes (33). Independent gene expression studies corroborated evidence for supranormal NFκB signaling, as well as Notch and p53/apoptotic signaling, in PMF (33, 77, 83). Evidence for NFκB signaling hyperactivation was also observed in *MPL* W515L model mice (84), and in *Jak2* V617F model mice with loss of *Dnmt3a*, which showed a myelofibrosis-like phenotype (85).

**Figure 2:**
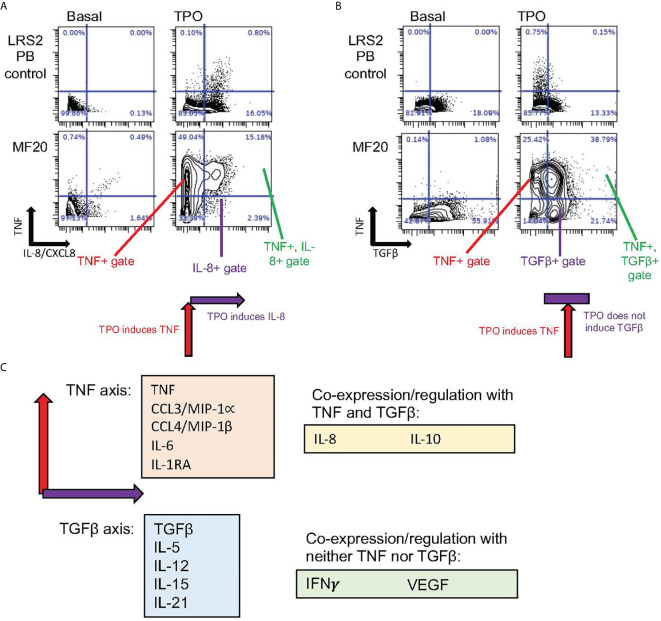
Distinct coexpressed groups of cytokines overproduced in MPNs. Data specific to MF, also utilized in Fisher et al. (48). **(A, B)** Presence or absence of tandem regulation of cytokine induction by TPO. Biaxial plots show in rows (from top to bottom) ex vivo cell samples from healthy control blood and blood from the *JAK2* V617F mutant MF patient MF20. Columns show cytokines as identified in cells by mass cytometry (CyTOF) after 4-hour incubation either without stimulation (Basal) or stimulated by TPO. **(A)** In monocytes, TNF (Y axis) showed coexpression with IL-8/CXCL8 (X axis) when induced by TPO. Combined induction is illustrated by schematic below lower right plot. **(B)** TGFβ (X axis) showed minimal basal coexpression with TNF (Y axis) in monocytes. TPO stimulation, however, induced TNF but not TGFβ, as illustrated by schematic under lower right plot. **(C)** Schematic depicting “axis” groups of cytokines overproduced in MF myeloid cells ex vivo. The majority of cytokines could be separated into TNF or TGFβ “axis” groups (related to biaxial plots in **A**, **B**), based on co-expression with either TNF or TGFβ after stimulation with TPO, TNF, or a TLR receptor ligand (R848 or PAM3CSK4), with Pearson R>0.25 (48). Of 15 cytokines assayed by CyTOF in Fisher et al. (48), only VEGF and IFNγ failed to demonstrate coexpression with either TNF or TGFβ, while IL-8/CXCL8 showed some evidence of coregulation with both (R>0.25).

Since MF patients frequently exhibit mobilization of CD34+ hematopoietic stem and progenitor cells (HSPC) from the bone marrow to the peripheral blood and spleen, it is possible to study cells occupying a spectrum of hematopoiesis from stem cells to more mature cells, using blood cells from MF patients, and comparing these to cells with comparable immunophenotypes in healthy control bone marrow and peripheral blood. Using the mass cytometry approach, our group was able to analyze cytokine production throughout hematopoietic cell populations *ex vivo* in MF patients versus healthy controls (48). Among cytokines surveyed by mass cytometry, a subset were identified that were inducible by TPO and TLR ligands, most of which were also inducible by TNF: these included TNF itself, IL-6, and IL-8/CXCL8, all previously implicated in MPN pathophysiology (48). This result suggests that *in vivo* overproduction of these cytokines is supported by combined JAK2 and NFκB signaling hyperactivations. Basally supranormal, or constitutively elevated, production of these cytokines in MF cells, was not invariably sensitive to suppression by *ex vivo* ruxolitinib. Instead, inhibitors of NFκB, MEK, and p38MAPK, were more effective than ruxolitinib at reducing basal levels of TPO/TLR/TNF inducible cytokines. Another subset of MF overproduced cytokines, notably including TGFβ and VEGF, did not show any responsiveness to TPO, TLR ligands, TNF, or ruxolitinib. Their basal levels, *ex vivo*, were likewise either unaffected or slightly elevated by inhibitors of NFκB, MEK, p38MAPK, or JNK. This second set of cytokines was not frequently co-expressed with the first set at the single cell level, while the TPO/TLR/TNF inducible cytokines were frequently co-expressed in individual MF monocytes (**Figure 2**). This suggests that overproduction of TGFβ and VEGF is directly driven by entirely different signaling pathways from TNF, IL-6, and IL-8/CXCL8, and which are separate from JAK-STAT, NFκB, MAPK, or JNK pathways.

Plasma cytokines elevated in the *MPL* W515L retroviral transplant mouse model of MF were also reduced by ruxolitinib, although not to the low levels observed in control mice (86). Cytokine levels were further reduced in this mouse model by BET bromodomain protein inhibitor JQ1, which reduced NFκB-associated gene expression, in combination with ruxolitinib (84). Since multiple cytokines are known to be inducible by NFκB, this pathway is an obvious candidate for a direct inducer of cytokine expression in MPNs.

Tgf-β mediated fibrosis in Tpo-treated mice has been linked to suppression of GATA-1 expression in megakaryocytes (87). However, the relationship of this model to human MF is uncertain, not only because it lacks a malignant clone, but also because it can be produced in NOD/SCID mice lacking a functional immune system and defective in cytokine secretion from monocytes (88); whereas monocytes, as well as megakaryoblasts, were observed to produce TGFβ in human MF (48). In contrast, pharmacologic inhibition of Aurora kinase A (AURKA) reduced plasma Tgf-β and bone marrow fibrosis in the *MPL* W515L retroviral transplant mouse model of MF (89). This result cannot be compared to those obtained in the same mouse model with ruxolitinib and/or JQ1, as Tgf-β was not among the cytokines assayed in those studies (84, 86). Therefore, the signaling mediators directly driving TGFβ and VEGF overproduction in MF remain unknown.

## TNF Is Implicated in Clonal Dominance in MPNs

**Figure 3:**
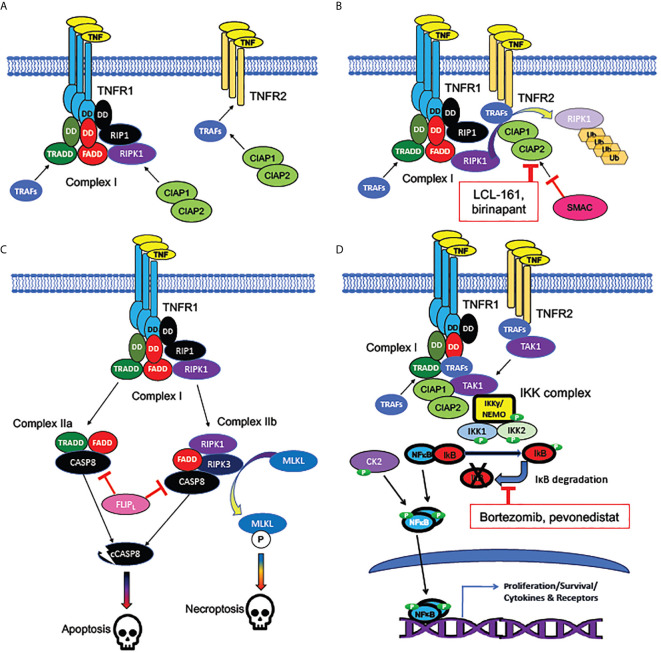
Signaling pathways activated by TNF receptors. **(A)** Bivalent cell-death regulating Complex I formed at plasma membrane by TNFR1 but not TNFR2. TNF receptors TNFR1 (encoded by *TNFSF1A*) and TNFR2 (encoded by *TNFSF1B*) are homotrimeric receptors binding homotrimeric TNF ligand. TNFR1, unlike TNFR2, contains an intracellular “death domain” (DD), which binds homologous DDs on intracellular RIP1, FADD, and TRADD, to compose the core of Complex (I) RIP1 recruits the pro-apoptotic RIPK1 kinase, while TRADD recruits the anti-apoptotic TRAF adaptor protein family members, which are necessary for activation of NFκB and MAP kinase signaling downstream of TNFRs. TNFR2, which lacks a “death domain”, can recruit TRAFs directly, but with a lower binding affinity than Complex (I) TRAFs can recruit CIAP1 and CIAP2, anti-apoptotic proteins which form heterodimers (encoded by *BIRC2* and *BIRC3*). **(B)** Mechanism of cell death inhibition by CIAPs at TNFRs. CIAPs are ubiquitin ligases, which polyubiquinate RIPK1, targeting it for degradation. CIAPs can be recruited intracellularly at TNF-bound TNFR1 or TNFR2. SMAC/DIABLO is an inhibitor of CIAPs: hence, SMAC/DIABLO mimetics (such as LCL-161 and birinapant) are pro-apoptotic. **(C)** Cell death pathways downstream of TNFR1. The uninhibited Complex I (with active RIPK1) promotes formation of the cytoplasmic death promoting Complexes IIa and IIb. Both complexes can activate cytoplasmic CASP8, the cleavage of which, to an active protease (cCASP8), initiates the apoptotic cascade. CASP8 activation can be inhibited by the long form of FLIP, encoded by the NFκB target gene *CFLAR*: a mechanism by which NFκB activation can promote cell survival. Complex IIb (containing RIPK3 as well as RIPK1) activates the kinase MLKL, which activates the necroptotic signaling cascade. **(D)** Activation of NFκB by TNFRs. TRAFs bound to TNFR1 or TNFR2 recruit TAK1, an essential activator of NFκB signaling. TAK1 activates the canonical NFκB pathway by recruiting the IKK complex, consisting of NEMO/IKKγ, IKK1, and IKK2. The IKK complex phosphorylates IκB family members, dissociating them from NFκB subunits and targeting them for degradation. NFκB, freed from IκB, is phosphorylated by both the IKK complex and casein kinase II (CK2), which in tandem activate NFκB subunits, which translocate to the nucleus to act as transcription factors. Bortezomib (directly) and pevonedistat (indirectly) inhibit IκB degradation. Among NFκB target genes are those encoding several cytokines and their receptors, as well as both antiapoptotic and pro-apoptotic components of the TNFR-NFκB signaling cascades.

MPNs are clonal diseases of the hematopoietic compartment, but the mechanisms for clonal expansion are not fully elucidated. Plausible hypotheses include that the molecular events driving pathogenesis, such as mutations that constitutively activate JAK-STAT signaling, either confer growth advantage to HSPCs compared to their wildtype counterparts under normal conditions, or can be protective against additional environmental factors related to aging, such as declining hematopoietic stem cell (HSC) functionality or inflammation (12, 54, 90). The TNF receptors TNFRSF1a and TNFRSF1b (also known as TNFR1 and TNFR2) activate different signaling pathways downstream of TNF binding (**Figure 3**), the former being associated with apoptosis and the latter with proliferation (81). Notably, TNFRSF1a, but not TNFRSF1b, contains an intracellular protein-protein binding domain known as a “death domain” (DD), which provides an essential scaffold for recruitment of the apoptosis-inducing multiprotein “Complex II”, or Death Inducing Signaling Complex (DISC), which can be activated by TNF (81, 91).

TNF can activate the canonical NFκB pathway, which is associated with myeloproliferation, both in myeloid neoplasms and in “stress” or “emergency” hematopoiesis following a hematopoietic insult such as the systemic inflammation resulting from an infection (92). Nonetheless, TNF has been observed to mediate substantial myelosuppressive effects by its direct action on hematopoietic stem and progenitor cells (HSPCs). Tnf can cause bone marrow failure and induce leukemic clonal evolution in mouse models of Fanconi anemia (93, 94). Furthermore, a recent study has shown that Tnf injected into normal, healthy mice is acutely toxic to myeloid progenitor cells and granulocytes, causing cell death by a combination of apoptosis and necroptosis, but that HSC are resistant to this Tnf toxicity (95). It has been hypothesized in several studies that malignant HSC from MPNs or leukemic initiating cells (LICs) in AML may harbor cell-autonomous mechanisms enabling these cells to further resist toxic or myelosuppressive effects of TNF acting on their non-malignant counterpart cells (53, 82, 96, 97).

The duality of TNF as an endogenous factor, which could potentially be either myelosuppressive or myeloproliferative, has led to hypotheses that this cytokine could play a major role in myeloid neoplasms, in the manner of promoting clonal dominance by exerting a myelosuppressive role on benign hematopoiesis while simultaneously exerting a myeloproliferative role on malignant hematopoiesis. This could be particularly crucial for chronic MPNs, where clonal dominance develops despite the malignant clone producing (mostly) functional mature myeloid cells. This feature of MPNs stands in contrast to AML or myelodysplastic syndromes (MDS), in which maturation-defective abnormal myeloid cells accumulate in a manner that can progressively crowd the normal hematopoietic niche out of existence. Therefore, a mechanistic hypothesis explaining the development of clonal dominance in MPN pathophysiology is necessary.

TNF is elevated in human MPN patient samples and is also elevated in *JAK2* V617F mouse models of MPN; notably, Fleischman et al. showed that PV, ET, and MF patients all had higher TNF levels in blood plasma than healthy controls, and that TNF levels correlated with *JAK2* V617F burden (53). Colony formation assays in methylcellulose revealed that whereas normal cells were inhibited by TNF, *JAK2* mutant cells were either resistant or stimulated by TNF (53). In colony assays from *Tnf* knockout mice with or without retroviral expression of *JAK2* V617F, the former clonal expansion observed in *JAK2* mutant cells in colony formation assays was limited. Additionally, in the mouse model, lack of Tnf did not prevent MPN development, but did severely limit the expansion of *JAK2* mutant cells. These data suggest that *JAK2* V617F HSPCs can both induce production of TNF and protect from its suppressive effects, thereby promoting clonal expansion (53). Simultaneously, the data suggest that TNF is not strictly necessary for development of an MPN disease phenotype. Recently, a study with induced pluripotent stem cell-derived CD34+ cells from a PV patient showed that *JAK2* V617F not only induces inflammation through IFNγ and NFκB pathways, but also protects from DNA damage due to inflammation *via* upregulation of dual-specificity phosphatase 1 (DUSP1) (98). The authors also observed that *JAK2* V617F-expressing cells only exhibited partial activation of ATM-related DNA damage checkpoint and p38/JNK stress pathway signaling under inflammatory conditions (98). In this system, expression of TNF, IFNγ or TGFß alone was insufficient for the induction of pro-fibrogenic chemokines CXCL9 and CXCL10, while the expression of TNF combined with IFNγ or all three cytokines produced a strong pro-fibrogenic response (98). By implication, a fibrogenic response could be produced by TNF plus IFNγ without artificial overexpression of TGFß, but none of the three cytokines alone sufficed to produce a strong fibrogenic response.

Clonal expansion mediated by TNF was reported in AML mouse models, showing that leukemic initiating cells (LICs) harbored constitutive NFκB activity due to an autocrine positive feedback loop with TNF (82, 97). NFκB signaling was bolstered by increased proteasome activation, which resulted in enhanced degradation of IκBα, a negative regulator of NFκB. This TNF/NFκB activity which increased LIC frequency in AML cells was not present in normal HSCs (82). Volk et al. also found that the administration of exogenous TNF had opposite effects with leukemic cells versus normal HSPCs in colony forming assays, where leukemic cells expanded and HSPCs were repressed (97), an analogous result to that obtained by Fleischman et al. with MPN HSPCs (53).

In 2011, a double knockout mouse for both *Tnfrsf1a* and *Tnfrsf1b* (encoding Tnfr1 and Tnfr2, respectively) was compared by Pronk et al. to either receptor knockout alone, showing for the first time that *in vivo* suppression of HSC cycling by TNF requires the expression of both receptors (99). A separate study in the same year showed that deletion of both Tnf receptors in mice provided a partial rescue of the combination of apoptosis and necroptosis produced in HSPCs by deletion of the Tgf-ß activated kinase (Tak1), which inhibits cell death pathways downstream of Tnfr1 (**Figure 3**) (100). While neither deletion of *Tnfrsf1a* nor *Tnfrsf1b* alone provided as strong of a rescue as the double knockout, the extent of rescue from *Tnfrsf1a* deletion was much greater than that from *Tnfrsf1b* deletion, consistent with Tnfr1 being the principal Tnf receptor mediating cell death (100).

In contrast to the result reported by Pronk et al. that both Tnf receptors *Tnfrsf1a* and *Tnfrsf1b* were required for Tnf-mediated repression of normal HSCs in mice (99), Heaton et al. observed that in MF CD34+ cells, selectively inhibiting TNFR2 but not TNFR1 was effective for blocking colony formation (96). In a *Jak2* V617F mouse model of MPN, blocking Tnfr2 (Tnfrsf1b) was sufficient to restore the expression of *Xiap* and *Mapk8* that was found to be downregulated in *Jak2* mutant vs wild-type cells. These genes were also found downregulated in MF CD34+ cells. These data suggest TNFR2 (TNFRSF1b) is likely to be an important cell-autonomous mediator of clonal expansion (96, 99).

Studies have shown that the accrual of secondary mutations, in addition to the primary drivers such as mutations in *JAK2*, *CALR*, or *MPL*, can be associated with disease progression and poorer prognosis. Mutations in *TET2* are among the most common non-*JAK2* mutations occurring in MPNs. Cells from *Tet2* knockout mice and *TET2* mutant human HSPCs were observed to have a growth advantage in clonogenic assays over non-mutant control cells (101). Chronic exposure to TNF in these cells led to myeloid skewing and increased resistance to apoptosis (101). A previous study from the same research group showed that macrophages deficient in TET2 were hyperinflammatory (102). Therefore, *TET2* mutations, like *JAK2* V617F, can lead to similar dual outcomes of promoting both an inflammatory environment and resistance to myelosuppressive effects of TNF, thus leading to clonal dominance of the mutant cells (102). Interestingly, other studies have shown that in MPN patients with *JAK2* V617F and *TET2* mutations, the presence of the *TET2* mutation in single cell-derived clones conferred an advantage towards clonal dominance, but not *JAK2* V617F on its own (103, 104). Kent et al. in 2013 studied highly purified *JAK2* V617F HSCs from a *JAK2* V617F mouse model with an ET phenotype, and observed that the mutation reduced HSC numbers, but that early progenitors exhibited increased proliferation and differentiation (105). This result suggests that while *JAK2* V617F confers hyperproliferation to the malignant clone within the myeloid progenitor population, it may be insufficient to establish clonal dominance at the level of HSC, and therefore that additional mutations or other pathogenic mechanisms may be necessary for clonal dominance to occur within the HSC population.

## NFκB Pathway Hyperactivation Is Systemic in MPNs and May Affect Stromal-Hematopoietic Interactions

NFκB signaling hyperactivation has been observed in multiple cancers, including lymphoid neoplasms (106), AML (107), MDS (108), and myelofibrosis (109). In contrast to lymphoid neoplasms, where mutations in NFκB pathway related genes are common, in myeloid neoplasms, mutations in NFκB pathway related genes are very rare (110). Since NFκB signaling is not (or very rarely) altered directly by mutation in myeloid neoplasms, it must be altered indirectly.

**Figure 4:**
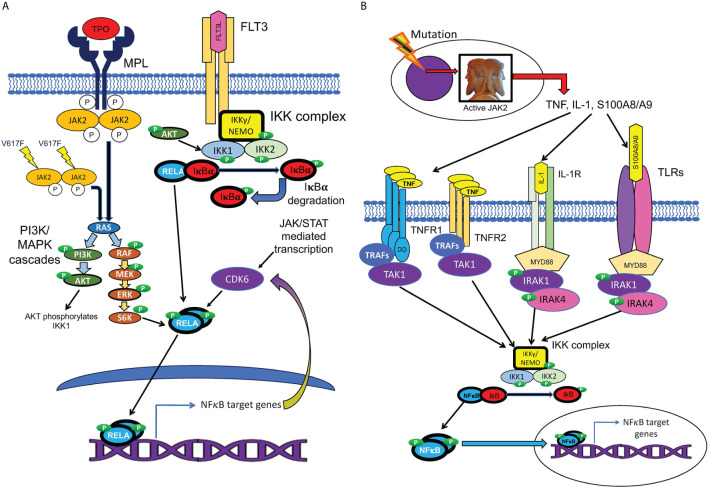
Cell-autonomous and non-cell-autonomous paths to NFκB pathway activation in MPNs. **(A)** Hypothesized pathways for cell-autonomous activation of NFκB downstream of active JAK2. JAK2 is activated by cytokine receptors such as MPL, or constitutively resulting from the V617F mutation. Active JAK2 activates RAS, in turn activating the MAP kinase and PI3 kinase signaling cascades, which are active in MPN HSPCs. Reported inputs of these pathways to NFκB include phosphorylation of IKK1 by AKT and phosphorylation of p65/RELA by S6 kinase (111, 112). The cell cycle kinase CDK6, a transcriptional target of both JAK/STAT and NFκB signaling, also can phosphorylate RELA (113). Active phosphorylated RELA translocates to the nucleus to mediate transcription. **(B)** Several non-cell-autonomous inputs overproduced in MPNs can activate NFκB. MPN driver mutations produce JAK2 hyperactivity, which leads to pathophysiologic production of TNF, IL-1, and TLR ligands S100A8/A9 and related family members. NFκB is activated either by TNFRs *via* TRAFs and TAK1, or by TLRs or IL-1 receptor, *via* the adaptor protein MYD88, which binds to these receptors, and recruits IRAK1/IRAK4 kinase heterodimers, which in turn activate the IKK complex.

The indirect constitutive activation of NFκB signaling (i.e. not *via* mutation of an integral NFκB pathway component) may occur by a combination of cell-autonomous and non-cell-autonomous mechanisms (**Figure 4**). NFκB signaling can be activated cell-autonomously by signaling downstream of an activating kinase mutation, such as *FLT3*-ITD or *JAK2* V617F. A mechanism has been described for FLT3 to activate NFκB signaling by direct binding to the IKK complex and consequent phosphorylation of IKK2 (114). A similar mechanism has been described where a Ras/PI3K/AKT pathway activated in AML cells led to activation of NFκB, which could be suppressed by pharmacologic AKT inhibition (111). Since the PI3K/AKT pathway, as well as NFκB, can be activated by TNF (97), this could be considered a feed-forward activation downstream of TNF receptors. Activation of ERK and downstream targets have been identified in individual MF and post-MPN sAML patients (33), and likewise JAK2-dependent ERK activity has been shown to contribute to disease phenotypes in *JAK2* V617F and *MPL* W515L expressing mice (112). The cell cycle activating kinase CDK6 is another potential activator of NFκB, as it shares with the IκB kinase family the activity of phosphorylating the NFκB subunit p65/RELA at serine 536, a phosphorylation necessary for activating it as a transcription factor (113, 115, 116). This would suggest the possibility that myeloproliferative HSPC could have higher intrinsic NFκB activity than quiescent HSPC, simply by virtue of higher CDK6 activity present in the cell cycle. In MPNs, this would presumably be an indirect consequence of the JAK2 hyperactivity responsible for the myeloproliferative phenotype. The role of CDK6 in MPNs is currently unclear: mice with *Cdk6* deletion along with *JAK2* V617F exhibited a somewhat less severe MPN phenotype than mice with *JAK2* V617F alone, but observed effects on NFκB target gene expression were ambiguous (117).

A prevalent alternative hypothesis is that in myeloid neoplasms, and particularly in MPNs, NFκB signaling is most often activated non-cell-autonomously by inflammatory mediators. The most prominent suspects include the known NFκB activating ligands IL-1α and β, which have been shown to be upregulated in some cases of AML (118) and MF (43)(note that circulating levels of IL-1 are normally undetectable), and which can promote myeloproliferation and HSC depletion in mice (119); endogenous toll-like-receptor (TLR) ligands, such as S100A8 and S100A9, which are overexpressed in MF CD34+ cells (48, 77); and, most of all, TNF, which is frequently overproduced in all chronic MPNs (53), as well as in a subset of AMLs, most often corresponding to the M4/M5 (myelomonocytic) FAB subclass (82). While circulating TNF is normally undetectable in healthy individuals (43), it is detectable in the plasma of almost all MF patients, and of many PV, ET, and sAML patients (33, 41, 48, 53). Likewise, while it is not yet clear what proportion of patients with any category of myeloid neoplasm feature NFκB signaling hyperactivation, our study using mass cytometry identified that, in CD34+ cells from the majority of MF patients studied, levels of phosphorylated, and hence active, NFκB subunit p65/Rela, were above the range observed in healthy controls (33).

NFκB signaling hyperactivation observed in MF and in sAML, however, was not confined to CD34+ cells, but rather was observed throughout both myeloid and lymphoid cell populations (33). This is consistent with a non-cell-autonomous etiology. In a study using mouse transplant models of MPNs with activated alleles of either *JAK2* or *MPL*, co-transplanted with wild-type mouse bone marrow cells, NFκB hyperactivation was observed in co-transplanted non-mutant hematopoietic cells as well as mutant cells (84, 86). Likewise in human MPNs, the total population of lymphoid cells is most frequently only partly derived from the malignant clone, as malignant hematopoiesis is typically myeloid-biased (78). The prevalent hypothesis is, therefore, that NFκB hyperactivation is transmitted from the malignant clone to the residual non-malignant hematopoietic cells and to bone marrow and splenic stroma *via* NFκB-activating cytokines.

**Figure 5:**
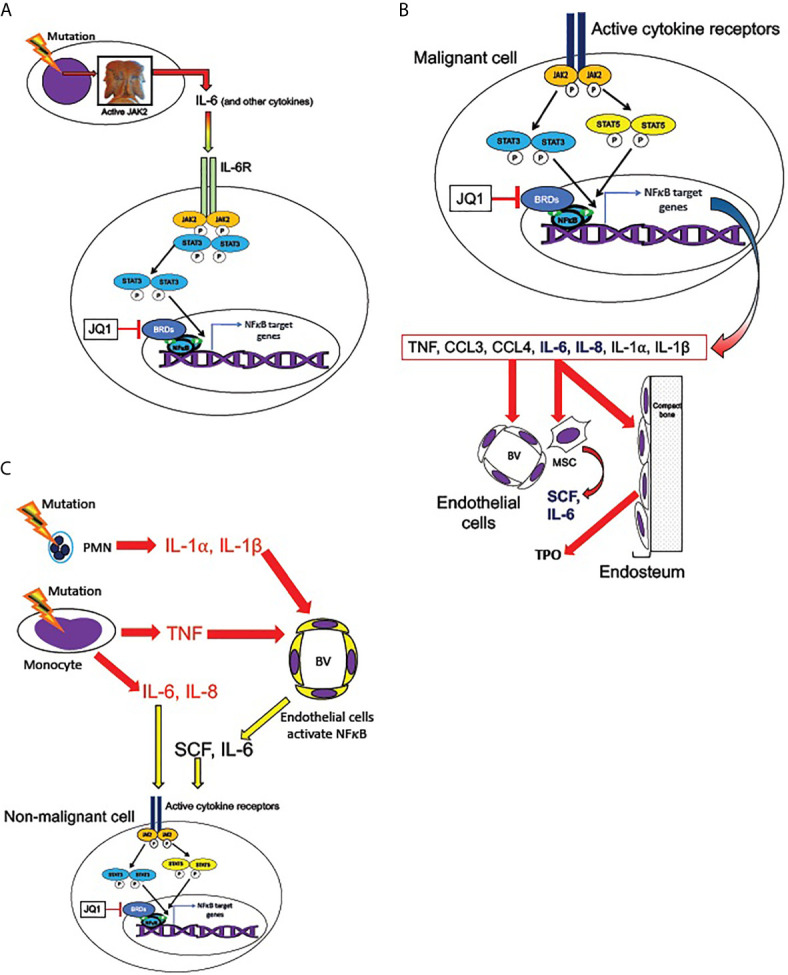
Transmission of JAK/STAT and NFκB pathway activation from malignant to non-malignant cells. **(A)** Malignant hematopoietic MPN cells transmit NFκB signaling activation to non-malignant cells, according to the hypothesized mechanism described by Kleppe et al. (86) MPN driver mutations produce hyperactive JAK2, leading to overproduction of cytokines, including IL-6. IL-6 receptor activation (in non-malignant cells) activates JAK2 and STAT3, which are required for maximal non-cell-autonomous NFκB activation in non-malignant cells. STAT3 shares multiple target genes with NFκB. NFκB mediated transcription requires BET bromeodomain proteins (BRDs) as cofactors, and therefore is subject to inhibition by the BRD inhibitor JQ1. **(B)** Active cytokine receptor signaling (such as from MPL or IL-6R) in malignant cells activates JAK2 and phospho-STATs 3 and 5, which co-activate multiple target genes along with NFκB. Among JAK2/STAT3,5 and NFκB co-induced targets are genes encoding several cytokines overproduced in MPNs: TNF, CCL3 (MIP-1α), CCL4 (MIP-1β), IL-6, IL-8, IL-1α, and IL-1β (41, 48, 53, 84, 86). IL-6 and IL-8 (bold) can activate JAK2/STAT3,5 signaling in non-mutant cells. These cytokines in turn act non-cell-autonomously on the endothelial cells of blood vessels (BV), mesenchymal stromal cells (MSC), and endosteal cells in the bone marrow. MSC are sources of SCF and IL-6 and endosteal cells are sources of TPO, which can activate JAK2/STAT3,5 signaling non-cell-autonomously (also see **Figure 6**). **(C)** Malignant neutrophils (PMN) in MPNs can produce IL-1α, and IL-1β, while malignant monocytes produce IL-6, IL-8, and TNF. TNF, IL-1α, and IL-1β, can activate NFκB signaling in endothelial cells of blood vessels (BV), leading them to release SCF and IL-6. These cytokines can combine with IL-6 and IL-8 secreted by malignant monocytes to produce activated JAK2/STAT3,5 signaling in both malignant and non-malignant cells.

Our group’s study of cytokine production by mass cytometry identified overproduction of several cytokines in MF monocytes and myeloid progenitor cells, which derive heavily from the malignant clone in most MF patients (78). Frequently overproduced cytokines in MF monocytes and myeloid progenitors included TNF and the ex vivo TNF-inducible cytokines IL-6, IL-8/CXCL8, CCL4/MIP-1β, and IL-1RA (48). This exact set of cytokines was previously shown to be overproduced in MF granulocytes (86). Likewise, these cytokines were among the larger set previously observed to be elevated in MF patient plasma (43). These cytokines represent a credible means for the malignant clone to induce NFκB hyperactivation and other signaling effects in the residual non-malignant hematopoietic and stromal cells (**Figure 5**).

Mouse homologs of MF-overproduced cytokines (except for IL-8/CXCL8, which does not have a direct homolog in mice) are overproduced in *JAK2* V617F (or *Jak2* V617F, if the mouse gene was mutated rather than the mutant human gene introduced) and *MPL* W515L MPN mouse models, and furthermore are also overproduced in non-mutant mouse cells co-transplanted with *JAK2* or *MPL* mutant cells: a non-cell-autonomous induction directed by the malignant cells, in direct analogy to human MPNs (86). Cytokine overproduction in MPN model mice was found to be heavily dependent on Jak2 phosphorylation of Stat3 (86), and on a maintenance of elevated NFκB-dependent gene expression by BET bromodomain proteins (84). In these studies, bone marrow fibrosis was also reduced by either *Stat3* deletion (86) or treatment with BET bromeodomain protein inhibitor JQ1 (84). Notably, the dependence of cytokine overproduction on Stat3 was only observed when all transplanted donor cells were *Stat3* null. In a co-transplant experiment with *MPL* W515L, *Stat3* -/- cells co-transplanted with wild-type mouse cells, cytokines were produced at levels similar to *MPL* W515L transplant recipients with intact *Stat3 (* 86 *).* Therefore, the requirement of Stat3 for cytokine production was found in non-malignant cells, in which cytokine production was induced non-cell-autonomously by the malignant cell population (**Figure 5**).

**Figure 6:**
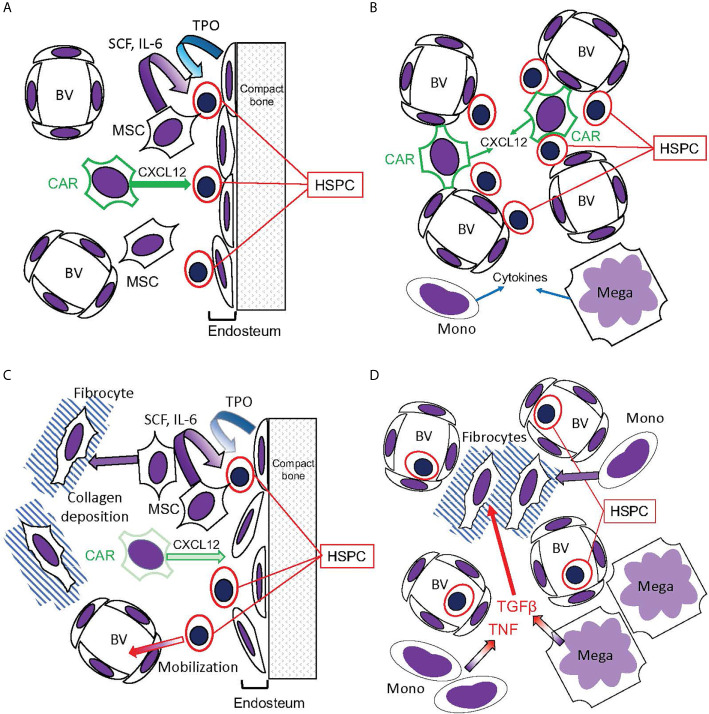
Hematopoietic bone marrow niches disrupted in MPNs. **(A**, **B)** Bone marrow niches in healthy hematopoiesis. **(A)** The endosteal niche: HSPC reside in contact with the endosteum, composed of osteoblasts that release TPO, promoting HSC quiescence. CXCL12 secreted by CAR cells promotes HSPC stasis in the bone marrow, while mesenchymal stromal cells (MSC) secrete SCF and IL-6. **(B)** The perivascular niche: HSPC reside in contact with blood vessels (BV), which are also contacted by the CXCL12-secreting CAR cells. This niche, however, is more prone to HSPC circulation than the endosteal niche. Monocytes (Mono) and megakaryocytes (Mega) secrete cytokines active on HSPC. **(C**, **D)** Disruption of hematopoietic niches in MF. **(C)** In MF, MSCs are abundant but CAR cells are reduced. The endosteum can be disrupted, and MSCs can differentiate into fibrocytes and deposit collagen, disrupting blood vessels in the hematopoietic space. Consequently, HSPC become mobilized. **(D)** Monocytes and megakaryocytes become abundant in the MPN bone marrow, releasing cytokines including TNF and the fibrogenic TGFβ. Monocytes, as well as MSCs, can differentiate into fibrocytes (71).

Much as the malignant clone can induce activation of NFκB signaling, and hence cytokine overproduction, even in residual non-malignant hematopoietic cells, it can also do the same to the non-hematopoietic stromal compartments of the bone marrow. Bone marrow stroma is a unique environment that supports normal hematopoiesis, known as the hematopoietic niche, which is severely disrupted by malignant hematopoiesis, particularly in MF and AML (120–123). In healthy bone marrow, HSCs are mainly localized to two distinct niches: the endosteal niche, characterized by direct HSC-osteoblast contact, and containing primarily quiescent HSCs; and the perivascular niche, characterized by direct contact of HSCs with endothelial cells, and containing the majority of proliferating, and potentially mobilized, HSCs and HSPCs (**Figure 6**). These niches both feature direct contact of HSCs and HSPCs with CXCL12-abundant reticular cells (CAR cells), which maintain HSPC localization in the bone marrow by producing CXCL12, which activates the receptor CXCR4 on HSPCs. Disruption of HSPC-CAR cell contact in MF may be one of the causes of the HSPC mobilization typically observed in MF. Mesenchymal stromal cells of the bone marrow (of which CAR cells are a subpopulation) are hypothesized to be the major fibrogenic cells in MF (124), although a role has also been described for monocyte-derived fibrocytes (71). Unquestionably, malignant hematopoietic cells can induce pathophysiologic changes in the non-malignant cells of the hematopoietic niche, *via* released cytokines and cell-contact-mediated factors.

**Figure 7:**
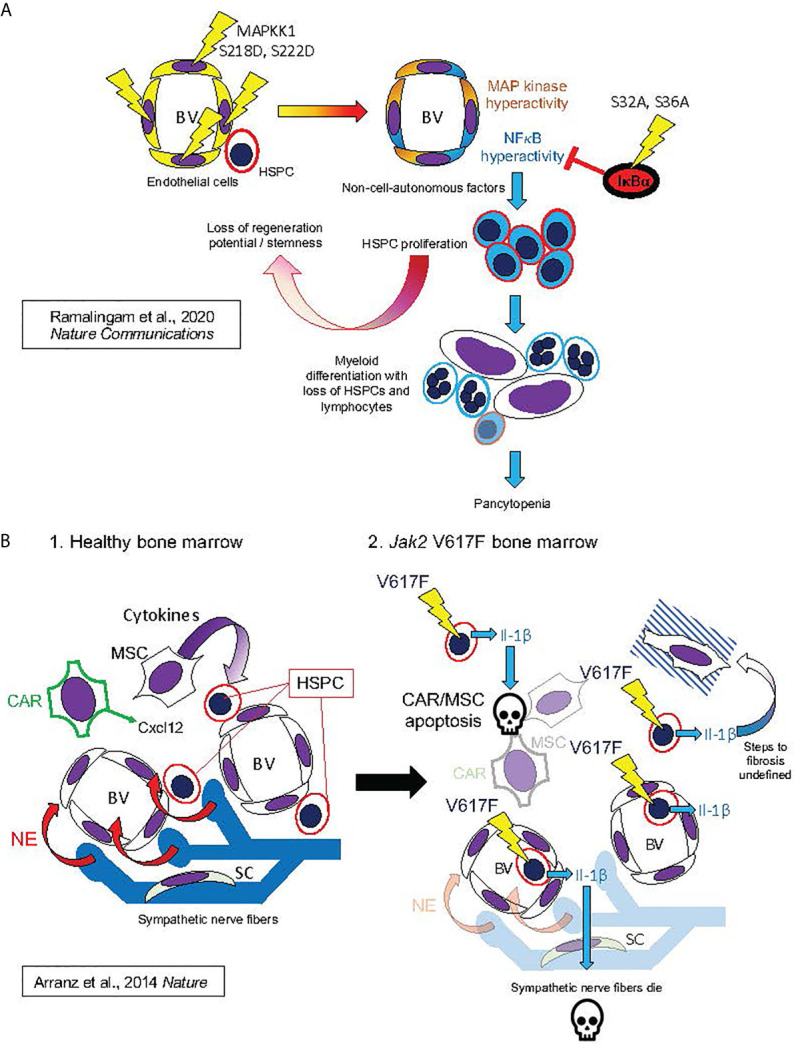
Hypothesized mechanisms of bone marrow niche remodeling in MPNs based on studies in mouse models. This figure illustrates mechanisms hypothesized from mouse model studies by Ramalingam et al. (127) *Nature Communications* (127) **(A)** and by Arranz et al. (128) *Nature* (128) **(B)**. **(A)** (from top left, following arrows indicating course of pathogenesis): Expression of MAPKK1 S218D, S22D mutant in endothelial cells, resulting in constitutive activation of MAP kinase signaling, also produced constitutive NFκB activation, possibly by a cell-autonomous mechanism, as described in **Figure 4A**. This led to HSPC proliferation and losses of stemness and regeneration potential in mouse HSPC: phenotypes derived non-cell-autonomously, since mutant MAPKK1 expression was confined to endothelial cells. HSPC phenotypes were dependent on NFκB hyperactivation in HSPC, as they could be entirely rescued by hematopoietic expression of the non-degradable IκBα S32A, S36A “super repressor” mutant. NFκB hyperactivation in HSPC promoted myeloid differentiation with loss of HSPCs and lymphocytes, resulting in pancytopenia and bone marrow failure (a phenotype also observed to result from pan-hematopoietic NFκB hyperactivation in mice) (129–133). **(B)** Bone marrow remodeling by hematopoietic *Jak2* V617F, analogous to human MPNs. 1. In healthy mouse bone marrow, Cxcl12 secreted by CAR cells and cytokines secreted by MSCs maintain HSPCs in the perivascular niche (analogous to **Figure 6B**). Bone marrow also contains sympathetic nerve fibers, which secrete norepinephrine (NE). Schwann cells (SC) are associated with the sympathetic neuronal fibers, and essential for their survival. 2. *Jak2* V617F, expressed in hematopoietic cells, causes secretion of Il-1β (Il-1α was not assayed). Il-1β caused apoptosis of CAR cells, other MSCs, and Schwann cells, leading to sympathetic denervation of bone marrow. The exact downstream signaling pathways to apoptosis and marrow fibrosis were not defined in this study. These features were, however, substantially rescued by either a catecholaminergic agonist or the natural Il-1 receptor antagonist Il-1ra, establishing essential roles of both Il-1 and sympathetic denervation in bone marrow pathophysiology caused by *Jak2* V617F.

Pathophysiologic intracellular signaling alterations in hematopoietic niche cells almost certainly produce reciprocal paracrine effects with malignant and non-malignant hematopoietic cells (**Figures 5** and **6**). Mesenchymal stromal cells in the bone marrow are a source for HSPC-promoting cytokines, including stem cell factor (SCF) and IL-6, and osteoblasts are a source for TPO, which promotes HSC quiescence in the endosteal niche (125, 126). NFκB activation in hematopoietic niche cells is likely to severely affect hematopoiesis. This hypothesis is supported by a recent study which used an inducible endothelial-specific expression system to produce constitutive MAP kinase signaling in endothelial cells, downstream of an introduced phosphorylation-mimic MAPKK1 S218D, S222D (127). This produced secondary NFκB signaling hyperactivation in the bone marrow endothelial cells (plausibly a cell-autonomous effect), and the hematopoietic phenotype of HSC depletion by induction of proliferation and differentiation of HSC to myeloid progenitors, and consequent preferential production of myeloid cells in the setting of overall pancytopenia (**Figure 7A**). This hematopoietic phenotype very closely resembles phenotypes observed when a phosphorylation-mimic Ikk2 (IKBKB) S177E, S181E was expressed pan-hematopoietically in mice, either heterozygously (129) or homozygously (130). It also resembles mouse phenotypes obtained when A20/TNFAIP3, an inhibitor of NFκB signaling activation by activated TNFR1, TLRs, and other signaling receptors, was eliminated (131–134). Furthermore, the hematopoietic phenotype induced by endothelial MAP kinase pathway hyperactivation was completely suppressed by introduction of IκBα (NFKBIA) S32A, S36A “super repressor”, which constitutively inhibits canonical NFκB signaling, exclusively in the endothelial cells (127). The implication is that NFκB hyperactivation in bone marrow endothelial cells, derived secondarily to MAP kinase pathway hyperactivation, was transmitted non-cell-autonomously to the hematopoietic compartment, resulting in NFκB-hyperactivated hematopoiesis. A reverse of this process may occur in MPNs, where NFκB signaling hyperactivation in hematopoietic cells can produce cytokine-mediated circular positive feedback with signaling hyperactivation in endothelial or mesenchymal components of the hematopoietic niche. Notably, inhibition of NFκB in mouse endothelial cells was also observed to improve hematopoietic recovery after myeloablative insults (135). This is further supporting evidence that inhibiting NFκB signaling may be a useful therapeutic modality to promote recovery of residual benign hematopoiesis.

Hematopoietic niche remodeling produced by JAK2 V617F has also been modeled in mice (**Figure 7B**). An inducible *JAK2* V617F mouse model (using *Mx1-Cre*, induced by poly-inosine-cytidine, a.k.a. poly-IC or PIPC) exhibited apoptosis of nestin-expressing mesenchymal stem cells (MSCs) and Schwann cells of sympathetic nerve fibers within the bone marrow, and consequent loss of sympathetic innervation (128). These pathophysiologic changes could be substantially prevented by 16-week treatment with either a catecholaminergic agonist or Il-1ra, an endogenous antagonist of Il-1 receptor, the human homolog of which, along with IL-1α and β, is also overproduced in human MF (43, 48). In the mouse model, the sequence of pathogenic events appeared to be: (1) secretion of Il-1β from hematopoietic cells (Il-1α was not assayed), (2) loss of catecholaminergic nerve fibers, (3) loss of *Cxcl12* expression in mesenchymal stromal cells, resulting in (4) mobilization of HSPCs from bone marrow, and finally (5) apoptosis of mesenchymal stromal cells (128). It is notable not only that a chain reaction of pathogenic events was unleashed non-cell-autonomously by *JAK2* V617F, but also that the second step in the chain, after JAK2 hyperactivation, was release of Il-1β, an NFκB activating ligand, from the hematopoietic cells. Therefore, an important role of NFκB in the remodeling of the bone marrow niche by MPNs is probable.

NFκB signaling in HSPCs is thought to be a mediator of “stress hematopoiesis”, or “emergency hematopoiesis”, in which HSPCs are activated to produce granulocytes, erythrocytes, and megakaryocytes, in the setting of sepsis or another extreme hematopoietic insult (92). MPNs can be considered to hijack the normal mechanisms of stress hematopoiesis to remodel the bone marrow niche in a manner deleterious to benign hematopoiesis, but to which the malignant clone is adaptable, and through which it thrives.

It is relevant to note that while NFκB signaling is clearly implicit in the pathophysiology of MPNs, its effects other than induction of cytokine production remain largely unknown. Moreover, not all NFκB signaling is equal in the context of myeloid neoplasms. NFκB can be activated by two major upstream signaling pathways, termed canonical and non-canonical NFκB signaling (115). It was found that a stabilized, non-degradable mutant of NFκB-inducing kinase (NIK), a crucial mediator of non-canonical NFκB signaling, prolonged survival of MLL-AF9 AML mouse transplant recipients (136). The effects of this mutation included a severe downregulation of nuclear Rela, a canonical NFκB subunit, which is elevated in and essential for MLL-AF9 AML (137), as well as upregulation of nuclear non-canonical NFκB subunits Relb, p50, and p52 (136). This is consistent with previous findings that canonical and non-canonical NFκB subunits can inhibit, as well as activate, transcription, depending on the exact DNA target sequence bound (115), and that Rela and Relb can mutually antagonize by the formation of inactive heterodimers (138). In MF CD34+ cells, while NFκB target genes were predominantly expressed at higher levels than in normal control CD34+ cells, a subset of NFκB-related genes, including *REL* and *NFKB1*, were observed to be downregulated in MF versus control CD34+ cells, suggesting a dichotomy in the pathologic dysregulation of NFκB signaling (33). This dichotomy, however, was not straightforwardly explicable as a divergence of canonical versus non-canonical NFκB signaling (33, 115).

## JAK Inhibitor Benefits and Limitations May Relate to Cytokine Mediated Inflammation

JAK inhibitors have demonstrated efficacy in reducing spleen volume and improving constitutional symptoms associated with MPNs, but the only curative therapy remains allogeneic stem cell transplant. Indeed, amelioration of splenomegaly was one of the principal aims targeted in the COMFORT trials of ruxolitinib for treatment of MF (139, 140). Ruxolitinib has been shown to reduce elevated levels of circulating inflammatory cytokines in MF, which may underlie the improvement in constitutional symptoms and splenomegaly observed with treatment (57). While ruxolitinib improves symptoms, it does not eradicate the malignant clone, induce molecular remission, or prevent transformation to acute myeloid leukemia (AML), and only shows modest survival benefit. Studies conducted in our laboratory indicated that ruxolitinib therapy reduces but does not rectify cytokine overproduction in MF (48). These findings may explain why certain disease features such as malignant clonal burden and marrow fibrosis remain refractory to ruxolitinib therapy.

Other JAK inhibitors besides ruxolitinib, namely momelotinib, pacritinib, and fedratinib, have demonstrated some ability to reduce cytokines overproduced in MF beyond that observed with ruxolitinib, possibly due to their inhibitory actions on other signaling molecules besides JAK2 (70, 141–145). Notably, both ruxolitinib and momelotinib inhibit JAK1 as well as JAK2, while pacritinib and fedratinib are relatively more specific for JAK2. JAK1 is activated by interferons, IL-3, IL-7, IL-6 and related cytokines, and IL-2 (86, 146). This means it could play a significant role in inflammatory responses to cytokines induced in MPNs. JAK1 overlaps with JAK2 in phosphorylating STAT3, which is necessary for signaling activated by multiple cytokines, and Stat3 is necessary for maximal cytokine production in *MPL* W515L model mice (86, 146). Loss of *Jak1* in mouse hematopoiesis causes accumulation of HSPCs, and blunted or absent proliferative responses to Il-3, Il-6, and type I interferons (84). Therefore, inhibition of JAK1 could potentially reduce both myeloproliferation and cytokine-mediated pathophysiology in MPN patients. Itacitinib, a JAK1 specific inhibitor, was studied in a Phase 2 trial of MF patients (142). Itacitinib produced reductions of constitutional symptoms and splenomegaly, but only modest or inconsistent reductions in plasma cytokines, or cytokines produced in MF myeloid cells *ex vivo (* 48, 142 *).*

Other relevant non-JAK targets of JAK inhibitors include FLT3 and Interleukin Receptor Associated Kinase-1 (IRAK1), targeted by fedratinib and pacritinib; JNK (also known as MAPK8 or SAPK1), targeted by momelotinib; and CSF1R, also targeted by pacritinib. IRAK1 is a Ser/Thr kinase activated downstream of IL-1 receptor and TLRs (see **Figure 4**). Active IRAK1 dimerizes with TRAF6 to activate the IKK complex, and hence, NFκB signaling. Implicitly, IRAK1 inhibition might reduce NFκB signaling in the presence the ligands of IL-1 receptor and TLRs. Likewise, CSF1R inhibition might mitigate monocytosis. IRAK1 inhibition has been proposed as a therapeutic strategy for myelodysplastic syndromes (MDS), as well as MPNs, based on preclinical studies (147). JNK/MAPK8 is a kinase that can be activated by TNF separately from NFκB signaling. It activates the AP-1 transcription factor, which shares a number of anti-apoptotic target genes with NFκB. Volk et al. (97) found that co-inhibition of JNK and NFκB signaling in *MLL-AF9* induced mouse model leukemic blasts could induce cell death (primarily necroptosis) in the presence of Tnf (97). Therefore, effects on several of the known targets of JAK inhibitors other than JAK2 could provide therapeutically useful effects.

Like ruxolitinib, other JAK2 inhibitors have also not demonstrated an impact on malignant clonal burden or reduction in bone marrow fibrosis. Ultimately, the consequences of cytokine overproduction in MF disease pathogenesis and the cellular pathways involved require further exploration in order to improve therapy for MPNs.

## Inflammatory Signaling Is a Basis for Novel MPN Treatment Modalities

The clearly prominent role of inflammation in the pathophysiology of MPNs has led to the hope that inflammatory signaling, either between or within cells, could be manipulated to lead to improved options for treatment. Prior studies have suggested links between TNF and clonal expansion, TGFβ and fibrosis, and IL-1 (α or β) and pre-fibrotic damage to the bone marrow milieu (53, 128, 148). The fibrogenic effect of TGFβ is likely to be separate from the pathogenesis of clonal expansion and myeloproliferation. A study using the *JAK2* V617F transgenic and *MPL* W515L retroviral transplant mouse models of MF showed improvement of bone marrow fibrosis and splenomegaly with galunisertib, an antagonist of the TGFβ receptor serine/threonine kinase ALK5, despite absence of any effect on any other hematologic parameter (148). Therefore, antifibrotic treatments, especially if targeting TGFβ or its downstream signaling, will likely need to be combined with anti-myeloproliferative agents such as ruxolitinib (149, 150), and/or agents for reducing malignant clonal burden, such as IFNα.

Alternate approaches for MF treatment could include targeting inflammatory signaling in the pre-fibrotic alterations of the bone marrow milieu or manipulating the inflammatory signaling abnormalities present in the disease to trigger cell death in the malignant clone. This latter approach is suggested by the known signaling pathways activated by the cytokine TNF (**Figure 3**). While TNF is an activating ligand for NFκB, MAP kinase, and JNK signaling, it also can activate a variety of cell death modalities: these are activated by a death-promoting signaling cytoplasmic complex known as Complex II (as opposed to the bivalent TNFR1 signaling Complex I), which is activated specifically by TNF binding to its receptor TNFR1 (TNFRSF1A), but not by the alternate TNF receptor TNFR2 (TNFRSF1B) (81). TNFR1, TNFR2, and members of the TLR/IL-1 receptor superfamily can all activate NFκB signaling, but only TNFR1 and several of its relatives, such as FAS/CD95, can activate Complexes I-II, which in turn activate signaling that can lead to apoptosis, necroptosis, or pyroptosis (81, 151). NFκB is known to activate transcription of a number of known anti-apoptotic target genes, including *BCL2* and *CFLAR* (encoding c-FLIP) (152). Therefore, it is hypothesized that inhibiting NFκB in the presence of TNF might promote cell death mediated by TNFR1 and Complexes I-II.

Etanercept, a dimeric recombinant TNFR2 (TNFRSF1B) extracellular domain fusion protein with human IgG1-Fc, is used clinically along with other TNF/TNFR antagonists as an anti-inflammatory agent, and was used as a single agent in a clinical trial for MF, resulting in improvement of constitutional symptoms, somewhat similar to observed effects of ruxolitinib (153). TNF/TNFR antagonism, however, does not exploit the principle of antagonizing anti-apoptotic effects of inflammatory signaling, while maintaining cell death inducing signaling intact.

Several basic studies in mice and human cells support a rationale for targeting NFκB activation downstream of TNFRs, with the goal of killing malignant HSPCs. Acute Tnf treatment in normal mice was observed to induce rapid cell death in the bone marrow, to which normal HSCs and Cd41+ megakaryoblasts were almost entirely resistant (with other primitive myeloid progenitors being partially resistant, in comparison with granulocytes), with these resistant populations being induced to enter the cell cycle, rather than being killed (95). The preservation of HSC was found to be dependent on the NFκB subunits p50 and p65/Rela, in absence of which HSC would succumb to a combination of apoptosis and necroptosis (**Figure 3**). This was coincident with more abundant nuclear p50 and p65/Rela in HSC than in other cells, and higher ratios of Tnfr2 to Tnfr1. Furthermore, human gene homologs of mouse HSC and GMP Tnf-induced gene expression signatures were found to be upregulated in human aging, MDS, and AML (95). Similarly, human MF but not healthy control CD34+ HSPC were found to be sensitive to reduction of colony forming activity by a TNFR2 (TNFRSF1B) blocking antibody, coincident with elevated expression of anti-apoptotic *BIRC2* and *BIRC3* (encoding cIAP1/2), and reduced expression of pro-apoptotic *XIAP* and *MAPK8* (encoding JNK), in MF HSPC (96). These results are similar to those of a study showing that combined inhibition of NFκB and JNK could promote apoptosis and necroptosis in AML blasts (97); although the role of JNK observed in the MF and AML studies was opposite (96, 97). Put together, the results of these studies suggest that HSCs, and MF HSCs in particular, may be dependent on TNFR2 signaling to NFκB, to rescue them from cell death, which would otherwise be induced by TNF through TNFR1.

Canonical NFκB signaling, in absence of an activating signal, is inhibited in the cytoplasm by the endogenous inhibitor IκBα, and signal activation by TNF triggers the degradation of IκBα (**Figure 3D**). Given this knowledge, therapeutic agents that could prevent the degradation of IκBα have been considered potential therapeutic NFκB inhibitors, including in the treatment of myeloid neoplasms. Bortezomib, a proteasome inhibitor with clinical activity against multiple myeloma, was able to inhibit both NFκB activation and bone marrow fibrosis in mice treated with Tpo (154). Phase I/II clinical trials of bortezomib for MF, however, showed no clinical benefit and significant toxicity (155, 156). There is no evidence currently suggesting specificity of bortezomib for IκBα.

A logically equivalent approach has been undertaken with another indirect NFκB inhibitor, pevonedistat, which inhibits the neddylating enzyme NAE, responsible for a covalent modification to cullin ring ligase enzymes necessary for the degradation of IκBα (157–160). Pevonedistat has been shown to synergize with TNF in promoting apoptosis of hepatoma cells (157), and has observed pro-apoptotic activity against AML, MDS, ALL, and lymphoma cells (158, 160–164). Pro-apoptotic effects of pevonedistat may be enhanced in combined treatment with other pro-apoptotic agents, such as BCL-2 antagonists or SMAC (second mitochondria-derived activator of caspase, also known as DIABLO) mimetics (162), or in combination with HDAC inhibitors (159). HDAC inhibitors, which can actually enhance NFκB activity by preventing deacetylation of p65/RELA, can provide apoptosis in combination with NFκB inhibition (165, 166).

We have also observed pevonedistat to inhibit production of TPO/TLR/TNF-inducible cytokines from MF patient myeloid cells *ex vivo*, supporting its potential as an anti-inflammatory agent for MF (48). Based on this hypothesis, our group has initiated a Phase I clinical trial, combining pevonedistat with ruxolitinib for MF treatment (NCT03386214). Phase I/II studies of pevonedistat in MDS and AML have shown tolerability, and a Phase III study of pevonedistat in combination with azacitidine in MDS/AML is currently ongoing (NCT03268954) (167–169). A caveat with pevonedistat is that, like bortezomib, its activity may not be specific to IκBα, and there is evidence that some of its pro-apoptotic activity may be independent of NFκB inhibition (162, 164, 170–173).

SMAC/DIABLO mimetics have recently been tested in pre-clinical and clinical studies for MF, with some encouraging results. SMAC inhibits endogenous inhibitor of apoptosis proteins (IAPs, also called BIRC family proteins). IAPs are ubiquitin ligases, which ubiquitinate the pro-apoptotic Complex I scaffold RIP1 and the non-canonical NFκB activating kinase NIK (NEMO-independent kinase), targeting these proteins for degradation (174). SMAC/DIABLO mimetics, by inhibiting IAPs, promote cell death in the presence of TNF or FAS (175–179). Two such compounds, birinapant and LCL-161, (**Figure 3B**) were observed pre-clinically to have an inhibitory effect on myeloid colony formation specific to MF, rather than healthy control, CD34+ HSPCs (96, 180). In a recently completed Phase II study of LCL-161, encouraging activity was observed, with an overall response rate of 32% (15/47) in patients with MF who were refractory or intolerant to JAK inhibitor therapy (174, 181).

Casein kinase 2 (CK2) phosphorylates RELA on serine 529 (S529). While RELA phosphorylation on S536 has been more thoroughly researched, we have observed that the two phosphorylation events were interdependent in the *JAK2* V617F mutant human erythroleukemia (HEL) cell line (33). Inhibition of CK2 with CX-4945 in human AML cell lines and primary AML CD34+ cells *ex vivo* was shown to induce cell cycle arrest, with downregulation of active RELA, AKT, and pSTAT3 (182). This represents a pharmacologic inhibition of NFκB that also might be adapted to MPN treatment.

Other potential therapeutic targets for anti-inflammatory therapy in MF include TLRs and their endogenous ligands, and IL-8/CXCL8, along its receptor CXCR2 and downstream signaling. Several endogenous TLR ligands of the S100 family were found to be overexpressed in MF versus normal CD34+ HSPC (33, 48, 77). Laouedj et al. showed that the ratio of S100A8 to S100A9 mediates a balance between immature cell proliferation, promoted by S100A8, versus myeloid differentiation, promoted by S100A9, in HOXA9-MEIS1 and MLL-AF9 AML mouse models (183). While both S100A8 and S100A9, as well as other related S100 isoforms, were found to be comparably overexpressed in MF versus normal CD34+ HSPC (48), altering the balance between them might prove to be therapeutically useful. There is not currently, however, a known pharmacologic strategy to manipulate the S100A8:S100A9 ratio, and indeed, the mechanism based on which high S100A8 expression and high S100A8:S100A9 ratio are poor prognostic indicators in AML remains unknown (183).

Overproduction of IL-8/CXCL8 was observed to be a poor prognostic feature in MF, particularly with respect to sAML transformation, and IL-8/CXCL8 is often highly expressed in MF CD34+ HSPC as well as monocytes (43, 48). High expression of its receptor CXCR2 was found to be an adverse prognostic indicator for survival in AML and transfusion dependence in MDS (56). CXCR2 activation by IL-8/CXCL8 was shown to activate MAPK/PI3K signaling pathways, leading to proliferative and pro-survival effects in AML CD34+ HSPC (56, 184). Therefore, any of IL-8/CXCL8, CXCR2, or downstream signaling, are plausible therapeutic targets for MPNs.

## Unanswered Questions in MPN Inflammatory Pathophysiology and Treatment

MPNs can be clearly described as systemic inflammatory diseases of the hematopoietic system, as well as neoplastic diseases. Despite substantial recent progress in understanding the inflammatory components of MPN pathophysiology, a number of unanswered questions remain, whose answering might contribute to substantial improvements in MPN treatment.

The first question concerns treatment modalities. JAK inhibitors counteract the primary molecular defect common among MPNs, and, while substantially improving quality of life for many patients, they only modestly impact clonal burden or long-term outcomes (68, 79, 185, 186). IFNα therapy, in contrast, can reduce malignant clonal burden in individual patients, despite having pro-inflammatory activity and substantial systemic inflammatory toxicity, resulting in poor tolerability for many patients (187–193). Why do these therapeutic agents have relative effects on clonal burden that are discordant with what might be intuitively expected based on their molecular targets? While there is some hope that a pro-apoptotic therapy with greater efficacy against the malignant clone than against residual benign hematopoiesis might be achievable, particularly leveraging differential responses to TNF and NFκB between malignant and benign hematopoiesis (53, 96), such therapies are only in their initial experimental stages, in clinical Phase I/II trials. It is not clear whether a pro-apoptotic or anti-inflammatory approach can be successful, even in combination with ruxolitinib, hydroxyurea, or IFNα. In some secondary MF patients, active hematopoiesis derives almost exclusively from the malignant clone. An important question is whether in this set of patients, restoring benign hematopoiesis is possible.

A second question derives from the evidence that the malignant clone effectively poisons the bone marrow microenvironment. In MPN mouse models, there is already evidence that this may be occurring in a step-wise manner, with sequential pathologic changes occurring in the bone marrow, with each step depending on one or more of those preceding it (128). Is there a sequential chain reaction of pathophysiologic events in human MPN disease progression that can be assaulted at its early stages to prevent (or, at minimum, make less likely) disease progression to MF or sAML? Determining this will require a great deal of further pathologic study of MPN bone marrow samples, including comparison of ET and PV to MF samples, early versus advanced stage MF samples, and ideally serial biopsies from individual patients. In a *JAK2* V617F mouse model, Arranz et al. showed, perhaps surprisingly, that loss of sympathetic innervation was a relatively early, and crucial, event in the pathologic remodeling of MPN bone marrow (**Figure 7B**): indeed, hematopoietic secretion of Il-1β was the only antecedent even in the chain reaction identified in their study (128). Is the progression of pathologic events similar or different in human patients? If it proves similar, this would be a strong case for anti-inflammatory therapy being applied not only to MF but also to ET and PV, on the theory that bone marrow inflammation represented an early event in the progression leading up to the bone marrow microenvironment becoming hostile to benign hematopoiesis while remaining receptive to malignant hematopoiesis.

A third question is whether similar, or different, therapeutics will be needed for different stages of disease progression. Blast phase, or sAML, may require cytoreductive chemotherapy, and lasting remissions from sAML have been rare (28, 40). If sAML remission is achievable, will either sAML in remission or MF ever be curable by means other than allogeneic hematopoietic stem cell transplant? And if so, would a therapy to eliminate a diminutive malignant clone in the case of minimal residual disease even plausibly be the same as what might eradicate a malignant clone in MF, which would usually contribute the majority, or even a near-totality, of hematopoietic cell production? It seems certain that these vastly different disease states would require substantially different treatment modifications, even if there were a common element to the otherwise diverse treatments. But would a common element be possible? A one-size-fits-all treatment is not likely to be effective for all stages of MPNs. Rather, there remains the hope that some selective treatment targeting the malignant clone might be useful at multiple stages, in conjunction with stage-specific treatments to be used in combination therapy – JAK inhibitors, anti-inflammatory agents, pro-apoptotic agents, and other signaling inhibitors – wherein might lie some hope for patients to be cured pharmacologically rather than exclusively by transplant.

## Author Contributions

All authors contributed to writing and editing the manuscript. Figures were prepared by DF and tables were prepared by JF. All authors contributed to the article and approved the submitted version.

## Conflict of Interest

The authors declare that the research was conducted in the absence of any commercial or financial relationships that could be construed as a potential conflict of interest.
